# Is low-protein diet a possible risk factor of malnutrition in chronic kidney disease patients?

**DOI:** 10.1038/cddiscovery.2016.26

**Published:** 2016-05-09

**Authors:** A Noce, M F Vidiri, G Marrone, E Moriconi, A Bocedi, A Capria, V Rovella, G Ricci, A De Lorenzo, N Di Daniele

**Affiliations:** 1 Department of Systems Medicine, Hypertension and Nephrology Unit, University of Rome ‘Tor Vergata’, Rome, Italy; 2 Haemodyalisis Service, ‘Nuova Clinica Annunziatella’, Rome, Italy; 3 Department of Biomedicine and Prevention, Division of Clinical Nutrition and Nutrigenomic, University of Rome ‘Tor Vergata’, Rome, Italy; 4 Nutrition Service, ‘Nuova Clinica Annunziatella’, Rome, Italy; 5 Department of Chemical Sciences and Technologies, University of Rome ‘Tor Vergata’, Rome, Italy

## Abstract

Chronic kidney disease (CKD) is becoming increasingly widespread in the world. Slowing its progression means to prevent uremic complications and improve quality of life of patients. Currently, a low-protein diet (LPD) is one of the tools most used in renal conservative therapy but a possible risk connected to LPD is protein-energy wasting. The aim of this study is evaluate the possible correlation between LPD and malnutrition onset. We enrolled 41 CKD patients, stages IIIb/IV according to K-DIGO guidelines, who followed for 6 weeks a diet with controlled protein intake (recommended dietary allowance 0.7 g per kilogram Ideal Body Weight per day of protein). Our patients showed a significant decrease of serum albumin values after 6 weeks of LDP (T2) compared with baseline values (T0) (*P*=0.039), whereas C-reactive protein increased significantly (T0 *versus* T2; *P*=0.131). From body composition analysis, a significant impairment of fat-free mass percentage at the end of the study was demonstrated (T0 *versus* T2; *P*=0.0489), probably related to total body water increase. The muscular mass, body cell mass and body cell mass index are significantly decreased after 6 weeks of LDP (T2). The phase angle is significantly reduced at the end of the study compared with basal values (T0 *versus* T2; *P*=0.0001, and T1 *versus* T2; *P*=0.0015). This study indicated that LPD slows down the progression of kidney disease but worsens patients' nutritional state.

Chronic kidney disease (CKD) represents a major problem of public health and its evolution in end-stage renal disease requires renal replacement therapy (hemodialysis, peritoneal dialysis (PD) or transplantation). Worldwide, CKD incidence is high.^1^ For example, CKD incidence, stages 1 to 4, in USA population (NHANES Study) is 13.1%. CKD is more frequent among older individuals and subjects with hypertension and diabetes, suggesting that CKD needs to have a central part in future public health planning.^2^ In 2007, average inpatient cost for the first month of dialysis in the US was $9846 per Medicare member and $22 841 per employer group health plan member.^3^ According to the United States Renal Data System database, in 2010, the total expenses per patient for hemodialysis was $87 561 compared with $66 751 for PD.^3^ Epidemiological data collected in Italy suggest that the prevalence of CKD in male subjects is 8.1% and in female subjects is 7.8%; these values are lower in comparison with European and non-European countries, probably because of the Mediterranean-style food.^4–7^ Thus, slowing the progression of CKD is necessary not only to improve the quality of life of uremic patients but also to reduce the significant impact that this disease has on public health costs.

In the last 100 years, CKD patient management of restriction of dietary daily protein intake, has a central role.^8^ During the 1960s, for the first time, Giovannetti and Maggiore^9^ set the stage for diet therapy treatments currently used in CKD patients. However, the role of low-protein diet (LPD) in the progression of CKD remains controversial with discrepancies between various studies performed.^8,10^ Despite the obvious benefits of protein restriction (such as the reduction of accumulation of nitrogenous wastes and metabolic disturbances, commonly associated with CKD) several studies have demonstrated that especially very LPDs could cause deterioration in the nutritional status of CKD patients.^10^ Menon and coworkers^11^ compared LPD with very LPD supplemented with keto acids and amino acids and demonstrated that both diets had no effects on the progression of kidney disease but increased the long-term risk of death.

In CKD patients, protein-energy wasting is a frequent condition, characterized by low serum levels of albumin or transthyretin, sarcopenia and weight loss. Protein-energy wasting is strongly related to mortality in CKD patients.^12^ A 10-year cohort study in 206 hemodialysis patients showed that serum albumin level was a stronger predictor of mortality, more than inflammatory markers or intima-media thickness of the common carotid artery.^13^ In CKD population, surrogates of over-nutrition, for instance obesity or hyperlipidemia, seem to be associated with increased survival; this phenomenon is now acknowledged as 'reverse epidemiology'.^14^

The aim of this study is to evaluate whether 6 weeks of low-protein diet has impact on body composition, inflammatory markers and progression of CKD in nephropathic patients (stage IIIb/IV according to K-DIGO guidelines).

Furthermore, we measured for the first time erythrocyte glutathione S-transferase (e-GST), a well-characterized cellular detoxifying enzyme, and erythrocyte catalase (e-CAT), a protecting enzyme against oxidative stress in CKD patients enrolled in LPD protocol.

In previous studies, e-GST was already considered a potential biomarker of blood toxicity and adequacy of the dialytic treatments in nephropathic patients.^15–17^

## Results and Discussion

All the enrolled 41 subjects completed the study, and all their data were eligible for the analysis. In Table 1 are summarized the demographic and clinical parameters of CKD patients at the enrollment into the study. As expected, we observed a statistically significant reduction, in all time-points, of creatinine and azotemia values, as reported in Table 2.

Moreover, we observed a significant decrease of serum albumin values after 6 weeks of LPD (T2) compared with baseline values (T0) (*P*=0.039). At the same time-points, total proteins were significantly lower at T1 (3 weeks of LPD) and T2 (6 weeks of LPD) with respect to T0 (*P*<0.0001) (Table 2). Inversely, C-reactive protein (CRP) at the end of the study increased significantly compared with start point values (T0 *versus* T2; *P*=0.0131).

Regarding lipid profile (total cholesterol, high-density lipoprotein cholesterol, low-density lipoprotein cholesterol and triglyceride), we observed changes statistically significant only for total cholesterol and low-density lipoprotein cholesterol; in particular, we measured a reduction at T1 for both parameters (Table 2), as previously reported.^18^

In addition, we found a significant lowering of albuminuria and azoturia in every time-point (Table 2). The systolic blood pressure decreased significantly at T1 and T2 time-points compared with enrollment time (T0), as reported in Table 2, but we did not observe any changes in diastolic pressure.

In Table 3, we summarized the body composition parameters observed in all time-points of the study. In particular, we underlined a significant reduction of anthropometric values of weight and body mass index (BMI) with respect to basal values (Table 3). In terms of body composition parameters, we saw a significant increase of total body water (expressed in % and in Liters) at T1 (3 weeks of LPD) and T2 (6 weeks of LPD) compared with the start values of the study (T0). Consequently, we detected a significant impairment of fat-free mass percentage at the end of the study (T0 *versus* T2; *P*=0.0489). These data are probably because of the increase of total body water observed.

The muscular mass, expressed in Kg and percentage, is significantly decreased after 6 weeks of LDP (T2) to baseline value (Table 3). Interestingly, we showed a progressive and significant reduction of body cell mass (BCM), in kg and in percentage, and BCM index (BCMI) at T0 *versus* T2 and T1 *versus* T2 (Table 3). These parameters are expression of metabolically active mass and consequently indirect indexes of malnutrition state of the patients. The ratio between extracellular mass (ECM) and BCM is used to identify fluid imbalance or malnutrition. In normal subjects, this parameter is in the range 0.85–1.00.^19^ The index cutoffs used to identify the malnutrition status of patients are: 1.1–1.3 for a moderate to poor nutritional condition and ≥1.3 for a very poor nutritional status.^20^ In our study, at baseline, the patients showed a mean value of ECM/BCM ratio of 1.1±0.4 indicating a poor nutritional condition. This value increased significantly at T1 (*P*=0.04) and at T2 (*P*=0.04) showing that LPD aggravated the malnourishment of CKD patients (Table 3).

The phase angle (PA) is significantly reduced at the end of the study compared with basal values (T0 *versus* T2; *P*=0.0001, and T1 *versus* T2; *P*=0.0015); this parameter is a negative prognostic factor for survival as previously demonstrated in the literature.^21,22^

**Figure 1:**
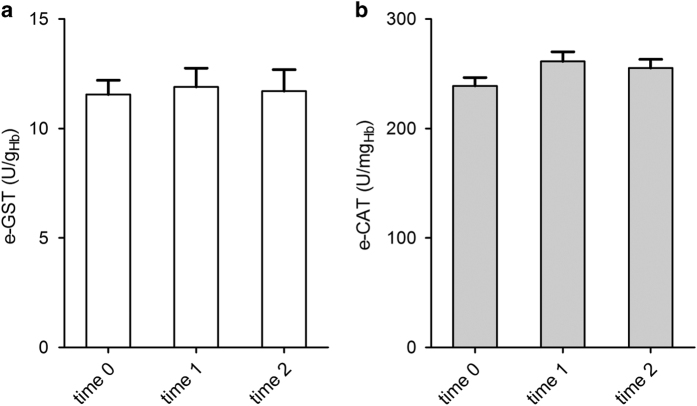
e-GST and e-CAT in patients during LPD. (**a**) e-GST after different time-points of diet treatment. Values are the mean of the measurements performed on 41 patients before diet (baseline) (time 0), 3 weeks of diet (time 1) and 6 weeks of diet (time 2). (**b**) e-CAT at different time-points of diet treatment. Values are the mean of the measurements performed on 41 patients before diet (baseline) (time 0), 3 weeks of diet (time 1) and 6 weeks of diet (time 2). Error bars are the S.E.M.

The e-GST levels (Figure 1a) and e-CAT levels (Figure 1b) remained stable during the experimental period, before starting the LPD till 6 weeks of LPD, in which patients were monitored for these enzymatic activities.

Although the LPD has been standardized as an important tool for the conservative therapy in CKD patients, currently there is a debate in literature about its real effectiveness.

In this study, we confirmed the benefits of this nutritional intervention in decreasing nitrogenous wastes and improving estimated glomerular filtration rate (GFR), as previous demonstrated.^23^ However, the protein restriction can be a further risk factor of malnutrition in these patients. In fact, we observed a significant reduction of biochemical parameters of nutritional status assessment such as serum albumin and total protein content.^24–26^ The reduction in serum albumin was observed only after 6 weeks of LPD because of its long half-life (about 20 days). Therefore, the reduced protein intake for the first 3 weeks showed no changes in the serum concentration of albumin.^25^

Traditionally, reduced serum albumin and decreased BMI are among the primary indicators of protein-energy malnutrition in uremic patients.^12^ In all time-points of our study, we observed a significant reduction of BMI.

The progressive malnutrition observed in CKD patients is associated with some markers of a chronic inflammation state that characterized the malnutrition-inflammation-atherosclerosis syndrome, inducing a vicious cycle where malnutrition, inflammation and atherosclerosis coexist.^27^ In this context, in addition to a deterioration of the nutritional status of the patients, we also found, after 6 weeks of LPD, a significant increase in inflammation parameters, evaluated by measuring CRP.

Our data suggest that LPD triggers a constant catabolic phase, which causes loss of protein from skeletal muscle. In fact, we observed a significant reduction of muscular mass at the end of the diet treatment. CKD *per se* is related to skeletal mass loss, causing the condition called ‘uremic sarcopenia’.^28–30^ The etiology of uremic sarcopenia is not well understood, although several factors (such as inflammation, hormonal unbalances, malnutrition and metabolic acidosis) are involved.

Another index linked to malnourishment state, assessed by bio-impedance analysis, is the PA.^22^ A substantial and significant decrease of PA was showed during patients' diet treatment. The PA is used as an indicator of cellular density; a low PA is associated to the damage of cell’s membrane and their impaired function. Actually, in literature, PA is negatively related to survival in several pathological conditions such as cancer, uremia and HIV infection.^31–33^ The PA is an independent predictor of survival in hemodialysis patients.^34^ An interesting study on PD patients showed that PA could be considered as an independent prognosis marker for survival and clinical improvement: the PD patients with PA>6.0° had higher survival compared with those with PA values lower than 6.0°.^21^ A PA reduction can reflect an increase in the ratio between extracellular and intracellular water or a decrease in BCM.^21^

In the CKD patients, the assessment of the fluid state such as total body water and extracellular water and the evaluation of BCM is crucial to verify the nutritional state. BCM represents the metabolically active cellular fraction of the body and, for this reason, it could be considered as a potentially sensitive indicator of lean tissue loss.^35^ In addition to BCM, lean tissue consists also in ECM. ECM/BCM ratio, which directly reflects the proportions between intracellular and extracellular space, is one of the most sensitive index of malnutrition.^20^ Avram *et al.*^20^ had demonstrated that an increased ECM/BCM ratio was considered a negative independent predictor index of survival in PD patients. Malnutrition features, characterized by a pronounced depletion of BCM, with a greater ECM/BCM ratio due to the lack of functional muscle mass, was observed, for example, in HIV patients and reported as an early marker of malnutrition.^36^

In this study, we also observed a significant decrease of systolic blood pressure (BP), related with the restriction of the sodium in the diet, according to guidelines maintaining sodium intake <2 g/day.^37^ It was demonstrated that arterial hypertension and CKD are closely associated. In fact, BP typically arises when renal function is compromised, and induces the progression of kidney disease.^38^ Experimental animal models have shown that arterial hypertension causes kidney damage and decreases the kidney's ability to remove sodium.^39^ High dietary salt intake not only exacerbates arterial hypertension in nephropathic patients, but also has the potential to directly aggravate kidney dysfunction. In a Cochrane database systemic review, it was demonstrated that on reducing salt intake in CKD patients, there was a significant decrease of systolic and diastolic blood pressure.^40^

Interestingly, e-GST and e-CAT levels remained stable during the period of the study. This is a strong indication that toxins and oxidative stress inducers did not increase during the LPD. The level of e-GST in all patients before the diet was high and corresponded to the one found previously in other CKD stage IV patients.^15,16,41^ This finding confirms a persistent blood toxicity status during the LPD even if a weak renal function improvement was achieved.

## Conclusion

In this study, we confirmed, coherently to the literature, that the LPD induces an improvement of the renal function, but at the same time, it contributes of the state of malnutrition usually observed in CKD patients.

The time limit of LPD did not allow us to evaluate a significant reduction of muscle mass, which is one of the pathognomonic criteria of uremic sarcopenia. Therefore, additional and prolonged studies will be needed to corroborate our hypothesis.

## Subjects and Methods

A total of 41 patients (22 males and 19 females), aged 73±14 years, were recruited from the Department of Internal Medicine, Hypertension and Nephrology Unit, ‘‘Tor Vergata’’ University Hospital and Haemodyalisis and Nutrition Service, ‘Nuova Clinica Annunziatella’, Rome (Italy) according to the level of kidney function (Table 1).

The selected patients had a mean GFR of 23±1 ml/min corresponding to stages IIIb/IV according to K-DIGO guidelines.^37^

The primary causes of renal failure were: chronic glomerulonephritis (32%), nephroangiosclerosis (58%), chronic pyelonephritis and interstitial nephropathy (2%), autosomal dominant polycystic kidney disease (2%) and other causes (~5%) (Table 1). Exclusion criteria were: corticosteroid treatment, cancer, pregnancy and nursing, and immunological or autoimmune diseases except type II diabetes. All patients were managed with standard diet conservative treatment (LPD) according to (Italian Society of Nephrology) SIN.^42^ Controls were carried out at baseline (T0), after 3 weeks (T1) and after 6 weeks (T2). All the subjects provided a signed consensus at the enrollment, according to a protocol previously approved by the ‘Tor Vergata’ University Medical Ethical Committee, Rome, Italy.

### Low protein diet (LPD)

In CKD, nutritional therapy has the aim to reduce intake of proteins, phosphorus, potassium and sodium, preserving an adequate energy intake. In CKD patients, the energy ingested is a crucial aspect of diet treatment. In fact, neutral or positive nitrogen balance normally requires adequate energy intake and poor energy intake may directly result in protein wasting.^11^ The daily energy intake is aimed at 30/35 kcal per kilogram ideal body weight per day.^8,9,42^ From the time of enrollment (T0) to the next 6 weeks, patients followed a diet with controlled protein intake (recommended dietary allowance 0.7 g per kilogram ideal body weight per day of protein) and low-phosphorus intake (8–12 mg/g of protein). The restriction of potassium intake varies according to blood values, usually about 8–17 mg/kg/day, sodium intake <2 g/day and calcium intake<2 g/day.

The macronutrient composition of the diet therapy was: carbohydrates, 50–60% kcal/day; proteins, 14% kcal/day; total fat, 30% kcal/day (saturated fat<7% kcal/day; polyunsaturated fatty acids, 10–20% kcal/day; monounsaturated fatty acids, 10–20% kcal/day; cholesterol consumption<300 mg/day), 15–20 g of fiber, folic acid 200–300 mcg. In the diet plan, we have used ‘non proteic’ (commercially available) carbohydrates in order to replace cereal foods (containing protein with low biological value) with similar products but with a low fat content and constantly controlled protein, sodium, potassium, tyrosine and phenylalanine. They also include inulin, a soluble dietary fiber. No alcoholic beverages were allowed.

The plan for each subject was obtained from a dietetic software package (Dietosystem, DS Medica, Milan, Italy).

### Anthropometric measurements

After 12-h overnight fast, anthropometric measurements were performed on subjects in underwear without clothes and shoes. According to standard methods, the body weight (kg) was measured to the nearest 0.01 kg, using an accurate balance scale (Invernizzi, Rome, Italy).^43^ Height (m) was measured using a stadiometer to the nearest 0.1 cm (Invernizzi), BMI was calculated according to Quetelet Index (calculated as body weight divided by height squared (kg/m^2^)).

### Bioelectrical impedance analysis

Resistance, reactance, impedance and PA at 50 kHz frequency were measured using bioelectrical impedance analysis (BIA 101S, Akern/RIL System, Florence, Italy).

The total body analysis allows the assessment of nutritional status and body fluids according to a bi-compartmental model. Bioelectrical impedance analysis is a safe, low-cost, noninvasive, rapid method for the assessment of body composition, displaying a great potential if used for epidemiological and clinical studies. Measurements were taken on left side of the body, with injection and sensor electrodes placed on the hand and foot in reference position.

Total body water, intracellular water, extracellular water, BCM, fat mass and fat-free mass are estimated using the manufacturer’s equations.^44^

### GFR estimation

The estimated GFR was calculated using the CKD epidemiology collaboration (CKD-EPI) formula.^45^

### Blood pressure measurement

Systolic and diastolic blood pressures were registered during each visit and defined as the average of two measurements 1 min apart, with 5 min rest before the first measurement.

### Analysis of blood samples

Early morning blood samples were taken from each patient for biochemical screening test after a 12-h overnight fasting. Blood samples were collected into sterile tubes containing ethylenediaminetetraacetic acid (Vacutainer, BD, Plymouth, UK), *via* venipuncture from the antecubital vein. All materials were immediately placed on ice and plasma was separated by centrifugation at 1600×*g* for 10 min at 4 °C. We measured serum lipid profile that included plasma total cholesterol, high-density lipoprotein cholesterol, low-density lipoprotein-cholesterol and triglyceride concentrations; for kidney function, we measured creatinine, azotemia and albumin, CRP, erythrocyte sedimentation rate, GST, e-CAT activity, uric acid and serum and urinary electrolytes (potassium, phosphorus, sodium, calcium, azoturia, sodiuria and albuminuria).

For determination of C-reactive protein, a nephelometric assay was used (BN IITM Nephelometer and PROTIS program, Simens Healtcare Diagnostic, Milan, Italy).

The lipid profile that included total cholesterol (TC), high-density lipoprotein cholesterol, low-density lipoprotein cholesterol and triglyceride was determined through standard enzymatic colorimetric techniques (Roche modular P800, Roche diagnostics, Indianapolis, IN, USA), according to the manufacturer’s procedures, with reagents provided by the same company.

All other parameters were analyzed according to standard techniques by the accredited Clinical Chemical Laboratories of the ‘Tor Vergata’ Policlinico (PTV) Rome, Italy.

### Chemicals and reagents

Glutathione, 1-chloro-2,4-dinitrobenzene, ethylenediaminetetraacetic acid, H_2_O_2_ and all other reagents were purchased from Sigma–Aldrich (St Louis, MO, USA) and used without further purification.

### Erythrocyte glutathione transferase (e-GST) activity

e-GST activity was determined with a spectrophotometric assay at 340 nm (37 °C), using an Uvikon 941 Plus spectrophotometer (Kontron Instruments, Watford, Herts, UK). Briefly, one volume (40 *μ*l) of whole blood was diluted in 25 volumes (1 ml) of bi-distilled water causing red blood cell hemolysis. After 2 min, 0.1 ml were diluted to a final volume of 1 ml containing 1 mM glutathione, 1 mM 1-chloro-2,4-dinitrobenzene in 0.1 M potassium phosphate buffer, pH 6.5 according to the standard procedure of Habig and coworkers.^46^ Results were expressed as enzyme units (U) per gram of hemoglobin (Hb) (U/gHb): one unit represents the amount of enzyme that catalyzes the conjugation of 1 micromole of glutathione to 1-chloro-2,4-dinitrobenzene in 1 min at 37 °C.^41^

### Erythrocyte catalase (e-CAT) activity

e-CAT activity was determined with a spectrophotometric assay at 240 nm (25 °C), using a Kontron Uvikon 941 Plus spectrophotometer (Kontron Instruments). One volume of 5 *μ*l of hemolyzed blood was diluted in 1 ml of potassium phosphate buffer 0.05 M pH 7.0 with ethylenediaminetetraacetic acid 0.1 mM, and finally 10 ul of H_2_O_2_ 1 M according to the standard procedure of Beers and Sizer.^47^ Results were expressed as enzyme units (U) per gram of Hb (U/gHb): one unit represents the amount of enzyme that catalyzes the decomposition of 1 micromole of H_2_O_2_ in 1 min at 25 °C.

### Statistical analysis

Data are represented as means±standard error of the mean (S.E.M.). All continuous variables were checked for normality using Kolmogorov–Smirnov test. Differences between baselines and final values were tested used paired samples *t*-test and Mann–Whitney test, as indicated The minimal level of significance of the differences was fixed at *P*≤0.05. Statistical analysis was performed using computer software packaged (SPSS for Windows version 13.0, SPSS, Chicago, IL, USA and Stata version 12.1, StataCorp, TX, USA).

## Figures and Tables

**Table 1:** Epidemiological parameters of chronic kidney disease (CKD) patients

*Patients characteristics*	
Number of patients	41
Gender (male/female)	(22/19)
Mean age (years)	73±14
Hypertension (%)	28 (68)
Diabetes mellitus (%)	11 (27)
Dyslipidemia (%)	12 (29)
	
*Likely underlying renal disease:*	
a) Chronic glomerulonephritis (%)	13 (32)
b) Nephroangiosclerosis (%)	24 (58)
c) Chronic pyelonephritis and interstitial nephropathy (%)	1 (2)
d) ADPKD^a^ (%)	1 (2)
e) Other causes (%)	2 (~5)

a ADPKD: Autosomal dominant polycystic kidney disease.

**Table 2:** Laboratory parameters and blood pressure at baseline (T0), after 3 weeks (T1) and 6 weeks (T2) of low-protein diet

*Laboratory findings*	*T0*	*T1*	*T2*	*T0 versus T1, P*	*T0 versus T2, P*	T1 *versus T2, P*
Creatinine (mg/dl)	3.3±0.5^a^	2.5±0.1^a^	2.3±0.2^a^	0.0001	<0.001	0.0002
eGFR^b^ (ml/min)	23±1^a^	26±2^a^	29±2^a^	0.0003	0.0019	<0.0001
Azotemia (mg/dl)	83 (43–207)^c^	74 (35–216)^c^	66 (37–182)^c^	0.0026	0.0017	n.s.
Albumin (g/dl)	4.1±0.1^a^	4.1±0.1^a^	4.0±0.1^a^	n.s.	0.039	n.s.
Total protein (g/dl)	7.3±0.1^a^	6.9±0.1^a^	6.9±0.1^a^	<0.0001	<0.0001	n.s.
CRP^b^ (mg/l)	1 (0–68)^c^	1 (0–66)^c^	2 (0–69)^c^	n.s.	0.0131	n.s.
Potassium (mEq/l)	4.6±0.1^a^	4.4±0.1^a^	4.5±0.1^a^	0.030	n.s.	n.s.
Phosphorus (mg/dl)	4.0 (3.0–5.8)^c^	3.8 (2.9–6.2)^c^	3.6 (2.5–5.5)^c^	n.s.	0.001	0.04
Sodium (mEq/l)	139±1^a^	138±1^a^	138±1^a^	n.s.	n.s.	n.s.
Calcium (mg/dl)	9.7 (7.8–11.0)^c^	9.6 (6.5–10.5)^c^	9.3 (7.4–10.6)^c^	0.0003	n.s.	0.0026
Total cholesterol (mg/dl)	177 (85–293)^c^	164 (91–335)^c^	164 (98–282)^c^	0.0025	n.s.	n.s.
LDL cholesterol (mg/dl)	104 (38–213)^c^	96 (41–238)^c^	102 (41–196)^c^	0.0118	n.s.	n.s.
HDL cholesterol (mg/dl)	51 (32–88)^c^	49 (30–90)^c^	47 (18–76)^c^	n.s.	n.s.	n.s.
Triglycerides (mg/dl)	133±7^a^	130±8^a^	144±10^a^	n.s.	n.s.	n.s.
Uric acid (mg/dl)	5.3 (1.5–12.1)^c^	5.6 (3.0–11.8)^c^	5.6 (3.3–6.9)^c^	n.s.	n.s.	n.s.
Albuminuria (mg/g)	51 (0–2237)^c^	33 (0–1799)^c^	22 (0–1656)^c^	<0.0001	<0.0001	0.03
Azoturia (mg/dl)	1088±98^a^	753±78^a^	603±67^a^	0.0001	<0.0001	0.01
Sodiuria (mmol/l)	61±5^a^	58±4^a^	61±4^a^	n.s.	n.s.	n.s.
Systolic blood pressure (mm Hg)	140 (105–224)^c^	130 (100–210)^c^	130 (100–185)^c^	0.0002	0.0006	n.s.

a Data are expressed as mean±S.E.M.

b eGFR, estimated Glomerular Filtration Rate; CRP, C-Reactive Protein. *P*<0.05 is considered statistically significant; n.s.=not significant.

c Data are expressed as median and range minimum-maximum.

**Table 3:** Body-composition parameters at baseline (T0), after 3 weeks (T1) and after 6 weeks (T2) of low-protein diet

*Body-composition parameters*^a^	*T0*	*T1*	*T2*	*T0 versus T1, P*	*T0 versus T2, P*	*T1 versus T2, P*
Weight (kg)	71.8±2.1^b^	70.6±1.9^b^	68.9±2.0^b^	0.0043	0.0044	n.s.
BMI (kg/m^2^)	27.8±0.8^b^	27.4±0.7^b^	26.6±0.7^b^	0.0052	0.0019	0.0367
RZ (Ω)	40 (24–81)^c^	38 (18–84)^c^	38 (20–72)^c^	n.s.	n.s.	n.s.
RX (Ω)	467 (254–609)^c^	457 (278–729)^c^	455 (255–599)^c^	n.s.	n.s.	n.s.
PA (°)	4.7 (3.1–8.8)^c^	4.8 (2.7–9.6)^c^	4.3 (3.0–7.6)^c^	n.s.	0.0001	0.0015
TBW (L)	38.1 (29.3–63.5)^c^	38.6 (28.9–65.3)^c^	38.8 (28.1–68.9)^c^	0.0167	0.0003	0.0012
TBW (%)	55.0±1.1^b^	56.0±1.1^b^	57.2±1.3^b^	0.0312	0.0468	0.0088
FFM (kg)	47.8 (14.3–85.2)^c^	49.9 (35.4–79.4)^c^	48.4 (34.7–83.1)^c^	n.s.	n.s.	n.s.
FFM (%)	68.5±1.7^b^	70.8±1.4^b^	72.1±1.7^b^	n.s.	0.0489	n.s.
MM (kg)	27.6 (20.3–57.0)^c^	27.9 (20.3–50.9)^c^	27.4 (20.0–47.9)^c^	n.s.	0.0005	0.0015
MM (%)	40.7 (28.5–72.4)^c^	40.5 (27.6–61.0)^c^	39.8 (29.8–54.9)^c^	n.s.	0.0280	0.0408
FM (kg)	22.7±1.5^b^	21.8±1.4^b^	19.9±1.4^b^	n.s.	n.s.	n.s.
FM (%)	29.7±1.5^b^	29.2±1.4^b^	28.0±1.7^b^	n.s.	n.s.	n.s.
BCM (kg)	21.8 (15.6–46.1)^c^	21.5 (15.0–48.8)^c^	20.4 (15.0–37.4)^c^	n.s.	0.0005	0.0055
BCM (%)	46.8±1.2^b^	46.1±1.3^b^	43.8±1.1^b^	n.s.	0.005	0.0046
BCMI (kg/m^2^)	8.9 (5.8–17.1)^c^	8.5 (5.6–15.3)^c^	7.9 (6.1–14.6)^c^	n.s.	0.0005	0.0008
ECM (kg)	25.4 (16.6–52.5)^c^	25.5 (16.3–39.2)^c^	24.6 (16.1–37.7)^c^	n.s.	n.s.	n.s.
ECM (%)	53.2 (26.5–66.4)^c^	52.7 (32.2–70.0)^c^	51.8 (38.2–66.7)^c^	n.s.	n.s.	n.s.
ECM/BCM	1.1±0.4^b^	1.3±0.4^b^	1.3±0.4^b^	0.04	0.04	n.s.

a BMI, body mass index; RZ, resistance; RX, reactance; PA, phase angle; TBW, total body water; FFM, fat-free mass; MM, muscle mass; FM, fat mass; BCM, body cell mass; BCMI, body cell mass index; ECM, extracellular mass. *P*<0.05 is considered statistically significant; n.s.=not significant.

b Data are expressed as mean±S.E.M.

c Data are expressed as median and range minimum-maximum.

## Abbreviations

CKD: chronic kidney disease
LPD: low-protein diet
K-DIGO: kidney- disease improving global outcomes
PD: peritoneal dialysis
NHANES: national heart and nutrition examination survey
e-GST: erythrocyte glutathione S-transferase
e-CAT: erythrocyte catalase
CRP: C- reactive protein
BMI: body mass index
BCM: body cell mass
BCMI: body cell mass index
ECM: extracellular mass
PA: phase angle
GFR: glomerular filtration rate
BP: blood pressure
SIN: Italian society of nephrology
CKD-EPI formula: chronic kidney disease epidemiology collaboration formula
TC: total cholesterol
S.E.M.: standard error of the mean
